# Robotic pancreas surgery for pancreatic cancer

**DOI:** 10.1097/JS9.0000000000000906

**Published:** 2023-11-21

**Authors:** Sarah B. Hays, Aram E. Rojas, Melissa E. Hogg

**Affiliations:** a Department of Surgery, University of Chicago, Chicago; b Department of Surgery, NorthShore University HealthSystem, Evanston, IL, USA

**Keywords:** Distal pancreatectomy, learning curve, pancreatic neoplasm, pancreatic surgery, pancreatoduodenectomy, robotic surgery

## Abstract

Since the introduction of robotic pancreas surgery in the early 2000s, there has been significant increase in the adoption of the robot to perform complex pancreatic resections. However, utilization of the robot for pancreatic cancer has lagged behind due to concern for inferior oncologic outcomes. Furthermore, research in this field has previously been limited to small, single institution observational studies. Recent and ongoing randomized controlled trials in robotic distal pancreatectomy and robotic pancreatoduodenectomy have aimed to address concerns regarding the use of robotic techniques in pancreatic cancer. Together, these studies suggest similar, if not improved, outcomes with a robotic approach, including shorter hospital stays, expedited recovery with less postoperative complications, and equivalent resection rates, when compared to the standard open approaches. Additionally, surgical training in robotic pancreas surgery is of equal importance for patient safety. This review summarizes the available literature on the efficacy and safety of robotic pancreas surgery for pancreatic cancer, with specific focus on robotic distal pancreatectomy and robotic pancreatoduodenectomy.

## Background

Highlights

- The use of minimally invasive approaches for pancreatic surgery is rapidly increasing.
- Robotic distal pancreatectomy has similar oncologic outcomes to laparoscopic and open approaches, but has been associated with shorter length of stay, as well as lower rate of postoperative morbidity and complications.
- The learning curve for robotic distal pancreatectomy has been reported to be between 10–40 cases.
- Minimally invasive distal pancreatectomy is now considered to be the preferred method for resection of distal pancreatic malignancies by most surgical oncologists.
- Similar oncologic outcomes have been found between robotic, laparoscopic and open pancreatoduodenectomy. Some studies have suggested improved LN harvest and R0 resection rates in robotic pancreatoduodenectomy.
- Robotic pancreatoduodenectomy is associated with shorter length of stay, lower rates of postoperative complications, and decreased risk of needing to convert to an open procedure.
- The learning curve for robotic pancreatoduodenectomy has been reported to be around 80 cases.
- Ongoing randomized controlled trials will hopefully provide high quality evidence regarding the impact of robotic pancreas surgery on long-term oncologic outcomes and overall survival in pancreas cancer.

Pancreatic cancer is the 12^th^ most common cancer worldwide, with nearly 500 000 new cases in 2020. It is the 7^th^ leading cause of cancer related deaths^1^. In the United States, the 5-year survival rate for pancreatic cancer is between 5 and 10%^2^. Pancreatic ductal adenocarcinoma (PDAC) comprises over 90% of all pancreatic malignancies^3^. Despite advancement in the medical and surgical treatment of pancreatic cancer, the lethality remains elevated compared to the incidence.

Historically, surgery for pancreas cancer has meant large, open operations associated with high rates of morbidity and mortality^4^. Advances in minimally invasive techniques have provided surgeons across specialties with the opportunity to expedite recovery and decrease postoperative pain and complications^5^. Furthermore, robotic platforms provide surgeons the benefit of binocular three-dimensional vision, wristed instruments, stabilization of tremor, reduced operator fatigue and improved ergonomics^6^. In robotic surgery, the surgeon sits at a remote console, not scrubbed at the patient’s side. The console has a computer interface that controls instruments that are attached to the “robot” and introduced through laparoscopic ports. Intuitive Surgical (Sunnyvale, California) dominates the market and is on its’ fourth generation product, the Da Vinci Xi ©. Medtronic (Minneapolis, Minnesota) has a first generation Hugo robot. Ever since the first robotic pancreatoduodenectomy was performed in 2001, followed by the first robotic distal pancreatectomy in 2003, surgeons have been eager to determine if similar improved outcomes can be seen in robotic pancreas surgery^6,7^. We will look at perioperative and oncologic outcomes in detail below.

The increased use of the robot for pancreatic resections dictated the need for evidence-based guidelines. The 2019 Miami International Evidence-based Guidelines on Minimally Invasive Pancreas Resection were developed by expert pancreatic surgeons, and established standards for minimally invasive pancreatic surgery (MIPS), including both laparoscopic and robotic approaches^8^. The strongest recommendation was that minimally invasive distal pancreatectomy (MIDP) for benign and low-grade malignant tumours should be considered over open distal pancreatectomy. For higher grade malignancies, more complex resections, and pancreatoduodenectomy, further research was deemed necessary^8^. These guidelines were updated in July 2023, when the The Brescia Internationally Validated European Guidelines on Minimally Invasive Pancreatic Surgery (EGUMIPS) were released. These 98 recommendations on laparoscopic and robotic pancreas surgery spanned 8 relevant domains including indications, patient selection, surgical techniques and training, with varying strength of evidence^9^. As utilization of the robot for pancreatic surgery continues to increase, relevant guidelines are essential.

Recent randomized controlled trials (RCT) investigating outcomes of MIPS have helped to define the role of these techniques in pancreas surgery. The “Minimally Invasive versus Open Distal Pancreatectomy” (LEOPARD) trial in 2019 was a Dutch multi-centre RCT that demonstrated the benefit of a minimally invasive approach to distal pancreatectomy. Of the 47 patients who underwent a minimally invasive distal pancreatectomy (MIDP), 42 had a laparoscopic distal pancreatectomy (LDP) and 5 had a robot-assisted distal pancreatectomy (RDP). Compared to the open distal pancreatectomy (ODP) cohort, the MIDP cohort was found to have a shorter time to functional recovery (4 vs. 6 days, *P*<0.001), shorter length of hospital stay (LOS) (6 vs. 8 days, *P*<0.001), lower estimated operative blood loss (EBL) (150 vs. 400 ml, *P*<0.001), and lower rate of delayed gastric emptying (DGE) (6% vs. 20%, *P*=0.04)^10^. The MIDP cohort had a longer operative time compared to the ODP cohort (217 vs. 179 min, *P*<0.001)^10^.

More recently, the “Minimally Invasive versus Open Distal Pancreatectomy for Resectable Pancreatic Cancer” (DIPLOMA) trial was published in Lancet in 2023^11^. This was an international, multi-institutional, randomized non-inferiority trial designed to address concerns regarding the oncologic safety of MIDP as compared to ODP in pancreatic cancer. The DIPLOMA trial demonstrated non-inferiority of MIDP compared to ODP regarding resection rates. There were equivalent rates of R0 resection (73% vs. 69%, p_non-inferiority_=0.039), median lymph node (LN) harvest (22 vs. 23 nodes, *P*=0.86), and intra-peritoneal recurrence (41% vs. 38%, *P*=0.45)^11^. Additionally, median time to functional recovery and LOS were comparable, as were one- and two-year survival rates. Notably, of the 131 patients who underwent MIDP, only 31 patients underwent RDP^11^. While both LEOPARD and DIPLOMA strongly support the use of MIDP, the limited number of patients who underwent RDP demands further work to fully investigate the true impact of the robotic approach.

Furthermore, there is a paucity of RCTs investigating the role of minimally invasive pancreatoduodenectomy (MIPD). A recent international survey showed an increase in MIPD from 29% to 45.7%; however, the responding population is likely biased towards surgeons who perform MIPD^12^. The American College of Surgeons’ (ACS) National Surgical Quality Improvement Program (NSQIP) database shows that 11% of PD are completed minimally invasively (7.7% robotic and 3.4% laparoscopic)^13^. Currently, three RCTs comparing RPD versus OPD have finished accruing, but the data has not yet been published. One is a single institution trial from Germany, another includes three institutions from China, and the most recent is multi-institution trial from multiple European countries, and includes laparoscopic PD. While we await the results of these trials. we largely rely on retrospective and prospective studies to assess the role of robotic surgery in pancreatic cancer.

## Robotic distal pancreatectomy

**Figure 1:**
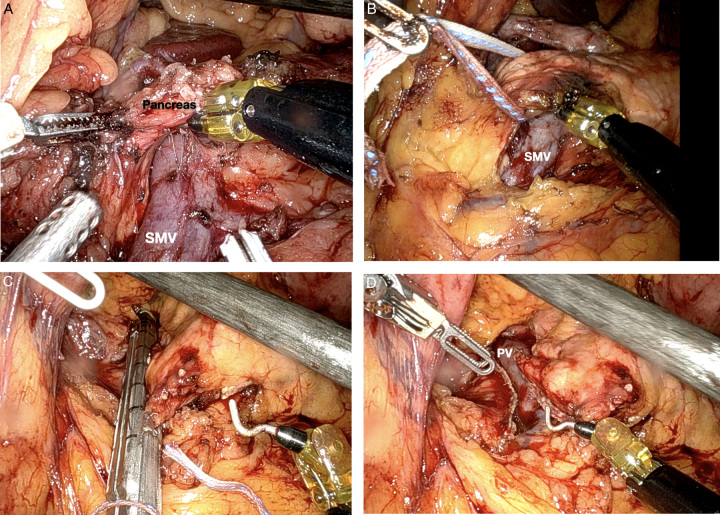
Robotic distal pancreatectomy dissection and resection. (A) Creation of the retro-pancreatic tunnel, with identification of the superior mesenteric vein (SMV) posteriorly. (B) Pancreas mobilization (hanging technique). (C) Transection of the neck of the pancreas with stapler. (D) Pancreas divided with visualization of the portal vein.

**Figure 2:**
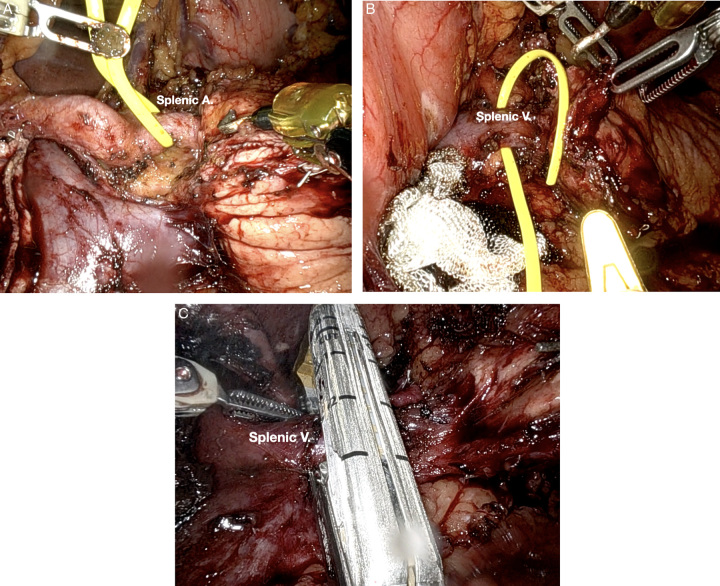
Robotic visualization of distal pancreatectomy vasculature. (A) Splenic artery in preparation for transection. (B) Splenic vein in preparation for transection. (C) Splenic vein transection with stapler.

The limited number of patients who underwent RDP in the LEOPARD (2019) and DIPLOMA (2023) trials reflects the current landscape of MIDP. The reality is that most surgeons are performing laparoscopic, not robotic distal pancreatectomies^14^. Still, there is a growing interest for broader implementation of RDP, largely due to the improved visualization and versatility of instruments, as well as the improved outcomes seen with other robotic procedures over laparoscopy^15^ (Figs. 1, 2).

### Oncologic outcomes

A prospective study from Memorial Sloan Kettering Cancer Center from 2000–2013 compared RDP (37) versus LDP (131) versus ODP (637) and demonstrated similar oncologic outcomes among the three groups, with high rates of R0 resection, from 88 to 100%. However, only 11% of the RDPs performed were for cancer. In this study, LN harvest was significantly higher in the ODP group (15 LN), compared to either the LDP (10 LN) or RDP group (12 LN) (*P*=0.04)^16^. A meta-analysis of two non-randomized controlled trials, including 21 patients who underwent RDP, drew similar conclusions regarding R0 resection and LN harvest^17^. Both studies included a small number of RDP, and thus it is difficult to derive practice shaping conclusions.

In a retrospective review of the National Cancer Database (NCDB) from 2010 to 2016 for patients with PDAC, Nassour *et al.* ^18^ found 332 patients who underwent RDP. The mean number of LNs examined was higher in the robotic approach (17 v. 15, *P*=0.002). Furthermore, RDP was associated with improved median overall survival (35.3 vs. 24.9 months, log-rank *P*=0.001), and accordingly higher rates of 1-year, 3-year, and 5-year overall survival^18^. RDP was also associated with higher rates of receiving adjuvant chemotherapy (64% vs. 56%, *P*=0.017)^18^. This is especially important as adjuvant systemic chemotherapy has been shown to improve survival in patients with surgically resected PDAC and is the standard of care^19,20^.

### Surgical outcomes

In a recent retrospective review using the ACS-NSQIP database, a matched analysis was performed of 2985 patients (1978 MIDP, 1007 ODP) and showed similar rates of major morbidity (8.65% MIDP vs. 9.76% ODP, *P*=0.37)^21^. MIDP was also associated with decreased LOS (5.5 vs. 7 d, *P*<0.001), but greater rates of postoperative pancreatic fistula (12.54% vs. 9.35%, *P*=0.02). Notably, this study did not differentiate between RDP and LDP within MIDP^21^.

However, another NSQIP study by Xourafas *et al.* ^22^ reviewed 1815 distal pancreatectomies and did differentiate between MIDP (921 ODP, 694 LPD, 200 RPD). Compared to ODP, the RDP group had shorter LOS (*P*<0.0001), lower rate of intra-operative blood transfusion (*P*<0.0001), and lower postoperative 30-day morbidity (*P*=0.0487), but longer operative time (*P*=0.003)^22^. Other reviews have found similar improvement in LOS, but no significant difference in EBL^18,23^. Xourafas and colleagues also reported lower rates of DGE and infection in RDP, but these results are not seen across all studies^22,23^. Aside from the Nassour and colleagues study discussed earlier, most studies report similar mortality rates across operative approach^18,21–23^.

### Laparoscopic approach versus robotic approach

While majority of the literature compares RDP to an open approach, when deciding between a laparoscopic versus robotic approach, it is equally important for a surgeon to know how the modalities compare to one another. In a meta-analysis of seventeen non-randomized observational clinical studies performed by Niu *et al.* ^23^, RDP was associated with longer operative time (*P*=0.01), but shorter LOS (*P*=0.03) and higher rates of spleen preservation (*P*=0.022), when compared to LDP. A propensity matched retrospective review compared 102 RDP to 102 LDP, and again found shorter LOS in the RDP cohort (7.67 vs. 8.58 days, *P*=0.032)^24^. Two studies did report a longer LOS in the RDP group, as compared to the LDP group. Chen *et al.* showed the RDP group had higher rates of R0 resection, higher LN yield, and more vascular resections, indicating that the RDP group underwent more extensive surgery, which may account for the longer LOS^25^. Additionally, Lof *et al.* ^26^ showed RDP was associated with improved rates of conversion, spleen preservation and readmission, to the detriment of longer duration of surgery and LOS. In a retrospective review of NSQIP data from 2010 to 2016, that included 196 RDP, operative time was the lowest in the RDP cohort (*P*<0.0001). While this is in contrast to the other studies reported, one possible reason is that the authors noted a trend toward more RDPs over the study period^27^. Thus, as surgeons become increasingly comfortable performing RDPs, operative time will likely continue to decrease.

An obvious limitation to a minimally invasive approach to any surgery is the need to convert to an open procedure, either due to failure to safely and efficiently progress through the operation, or in an emergency. Notably, the conversion to open rate in RDP has been shown to be significantly reduced when compared to LDP, a trend seen across multiple studies^22,24,25,27–29^. In fact, LDP was found to be an independent risk factor for conversion, which was associated with worse outcomes than initially planning to do an open surgery, including higher rates of DGE and possibly increased mortality^28^.

### Learning curve for robotic distal pancreatectomy

With the implementation of any new surgical technique comes unfamiliarity and increased risk to patients. Accordingly, significant effort has been dedicated to understanding the learning curves for robotic procedures. The learning curve of an operation refers to the time and number of operations it takes for a surgeon and/or an institution to become proficient^30^. A recent review of the learning curve literature found that a surgeon is judged to have reached the learning curve in pancreatic surgery based on operative time, EBL, complication rate, and LOS^31^.

Napoli *et al.* ^32^ calculated the learning curve of RDP on 55 consecutive patients based on reduction in operative time and found that the learning curve was achieved after 10 operations. After surpassing the learning curve there was also noted to be higher LN yield^32^. However, operative time is not the only important factor for surgeon proficiency. In a larger study by Shakir *et al.* ^33^, 100 patients underwent RDP, and significant reductions in operative time were seen after both 20 and 40 cases (331 vs. 266 vs. 210 min, *P*<0.0001). Likelihood of readmission decreased significantly after 40 cases, and there was a trend towards lower incidence of major morbidity, pancreatic leak, and shorter LOS^33^. In a systematic review of six studies on the learning curve on RDP, including the two studies mentioned previously, the learning curve was found to be 15 (range 5–37)^31^.

Surgeon learning curve is not the only important factor. A recent international, multi-centre, retrospective cohort study investigated the learning curve on MIDP performed from 2006 to 2019^34^. This study uniquely focused on MIDPs performed at centres that completed more than 15 distal pancreatectomies annually and had completed over 50 MIDP. The primary outcome was textbook outcome, a composite measure that reflects optimal operative outcome and an uneventful hospital course^34^. Overall, the learning curve for textbook outcome was estimated to be 85 cases. The learning curve was shorter for secondary outcomes, including 56 cases for reduction in operative time, 71 cases for decreased blood loss and 40 cases for decreased conversion rate^35^. It is important to note that this study combined LDP and RDP, so the described learning curves do not represent that of RDP alone, which may contribute to the longer learning curves reported.

Even with level one data and international consensus favoring MIDP, it is important to remember that every procedure has a learning curve for surgeons and for institutions. Programs performing these procedures should have adequate volume, adequate training, be pancreatic centres of expertise, and have all the capabilities to rescue their patients and track outcomes^36,37^. Most published series likely reflect a selection bias of high-volume centres; however national datasets can be a better snapshot of what is happening at all participating centres.

## Robotic pancreatoduodenectomy

In contrast to MIDP, the adoption of minimally invasive pancreatoduodenectomy (MIPD) has been much slower, likely due to initial resistance towards the procedure. Furthermore, a laparoscopic pancreatoduodenectomy (LPD) is a technically challenging procedure, limiting the use of MIPD prior to introduction of the robot. Still, as the demand for minimally invasive procedures increases across general surgery, so does demand for MIPD, with an increase from 12.2% of all pancreatoduodenectomies in 2010 to 21.4% in 2015^38^. National studies suggest that this growth is associated with a growing proportion of these cases being performed robotically and an increase in the number of centres offering robotic pancreatoduodenectomy (RPD)^39^.

**Figure 3:**
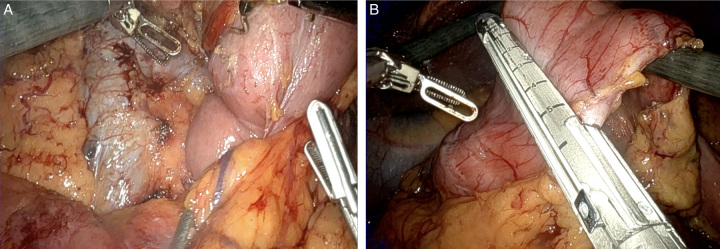
Robotic pancreatoduodenectomy retroperitoneal dissection. (A) Robotic Performing the Kocher Manoeuvre. (B) Transection across the distal stomach.

**Figure 4:**
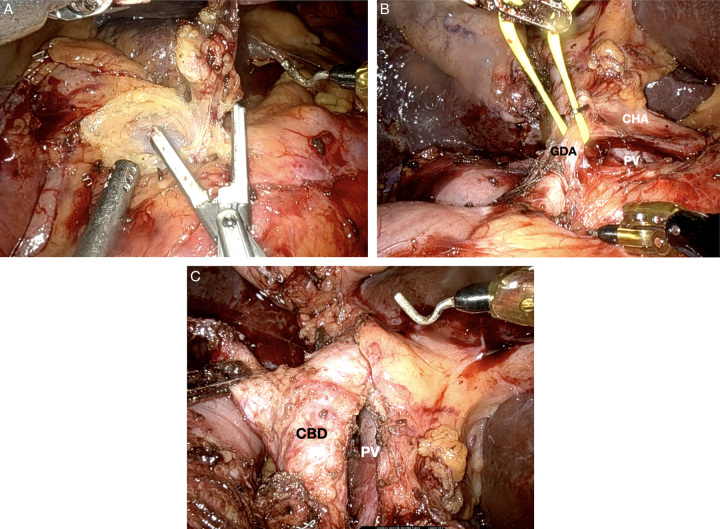
Robotic pancreatoduodenectomy portal dissection. (A) Dissection of the hepatic artery lymph node. (B) Dissection of the common hepatic artery (CHA) with the portal vein (PV) visualized posterior to the artery and identification of the gastroduodenal artery (GDA), in preparation for transection. (C) Dissection of the common bile duct (CBD), in preparation for transection, with identification of PV.

**Figure 5:**
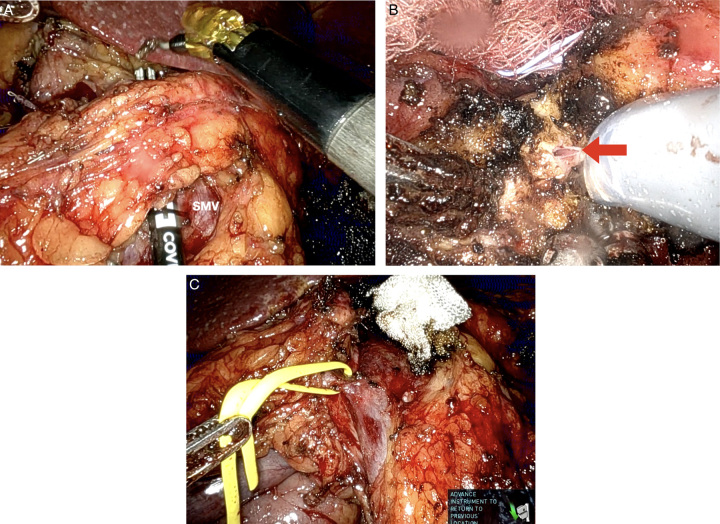
Robotic pancreatoduodenectomy pancreatic resection. (A) Creation of the retro-pancreatic tunnel, with identification of the superior mesenteric vein (SMV) posteriorly. (B) Transection of the neck of the pancreas with identification of the pancreatic duct (arrow). (C) Gastroepiploic vein.

**Figure 6:**
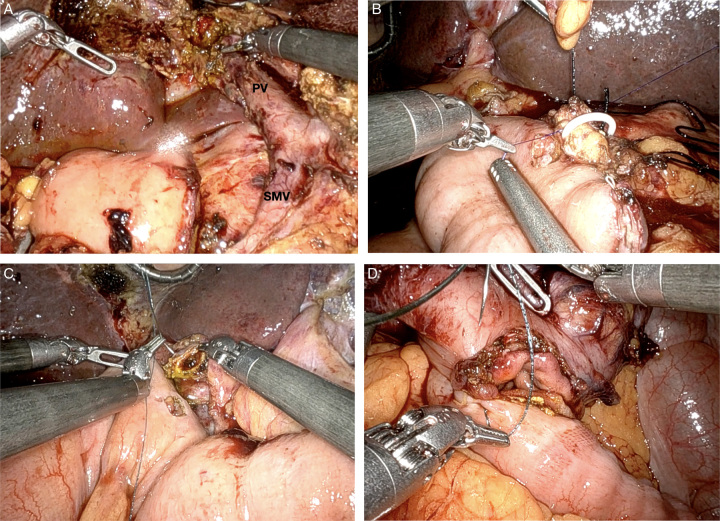
Robotic pancreatoduodenectomy reconstruction phase. (A) Identification of the confluence of the superior mesenteric vein (SMV) with the portal vein (PV). (B) Creation of the pancreatojejunostomy with a stent within the pancreatic duct. (C) Creation of the hepaticojejunostomy, starting with a corner stitch at 9 o’clock. (D) Creation of the gastrojejunostomy.

There are three major RCTs that investigated the role of LPD and have served as a basis for RPD. The “Randomized Clinical Trial of Laparoscopic versus Open Pancreaticoduodenectomy for Periampullary Tumours” (PLOT, 2017) and the “Comparison of Perioperative Outcomes Between Laparoscopic and Open Approach for Pancreaticoduodenectomy” (PADULAP, 2018) trial showed equivalent R0 resection rates, LN harvest and short term outcomes between open pancreatoduodenectomy (OPD) and LPD^40,41^. PADULAP demonstrated lower rates of postoperative complications in the LPD group^41^. In a majority of studies comparing LPD to OPD, there is no difference in mortality rates between the two approaches. However, the “Laparoscopic versus Open Pancreatoduodenectomy for Pancreatic or Periampullary Tumours” (LEOPARD-2) trial was terminated prematurely in 2019 for a difference in 90-day mortality (10% (5/50) in LPD vs. 2% (1/49) in OPD), *P*=0.2)^42^. Although this difference was not statistically significant, this was the largest RCT comparing LPD to OPD and sparked concern. Randomized trials investigating RPD remain ongoing, thus the data is confined to retrospective reviews and meta-analyses (Figs. 3 – 6).

### Oncologic outcomes

Hesitancy to adopt RPD has largely been driven by concern over worse oncologic outcomes compared to OPD, however this is not borne out by the data. In a NCDB retrospective review of 17,831 pancreatoduodenectomies, of which 626 were performed robotically, there was a higher average number of LNs examined in the RPD cohort compared to OPD^18^. Multiple single centre retrospective studies have also shown equivalent to improved LN harvest in RPD^43–45^. Furthermore, RPD and OPD have been shown to yield equivalent R0 resection rates^44,46^. One meta-analysis that included 2,175 RPDs even found that resection margin involvement rate was significantly lower in the RPD population compared to OPD^47^. The aforementioned NCDB retrospective review also found similar median overall survival between RPD and OPD (22 vs. 21.8 mo, log-rank *P*=0.755), a trend that has been confirmed in multiple single centre retrospective studies^18,43–46^.

### Surgical outcomes

Compared to OPD, RPD is almost unanimously associated with increased operative time^44,48,49^. Only one study, performed at a high-volume single institution, found longer operative time in the OPD cohort^50^. Notably, in this study, all RPDs and OPDs were performed by high-volume pancreatic surgeons who were beyond their learning curve in both procedures. This suggests that as the utilization of RPD increases and surgeons surpass their learning curve, RPD operative time may decrease, and this may no longer be a benefit of OPD. Similar to RDP, RPD is associated with lower EBL compared to OPD^44,48,49,51,52^. RPD patients were also found to have equivalent to shorter LOS, compared to their OPD counterparts^44–46,49,52,53^.

This improved LOS seen in RPD patients may be due to the reduced rate of complications associated with RPD. An ACS-NSQIP database analysis of 498 RPD and 12,612 OPD showed that patients who underwent RPD were less likely to have any complication (46.8% vs. 53.3%, *P*=0.004) or a surgical complication (42.6% vs. 48.6% *P*=0.008)^54^. However, in a subgroup analysis of those patients who underwent pancreatoduodenectomy for PDAC, the only significant difference was lower EBL in the robotic cohort. A single centre retrospective study from China compared RPD versus OPD for pancreatic neoplasm and again found lower incidence of Clavien-Dindo III-V complications (14.7% vs. 28% *P*=0.042) in the RPD group^51^.

Major complications following pancreatoduodenectomy include postoperative pancreatic fistula (POPF), post-pancreatectomy haemorrhage (PPH), DGE and wound infection. An ACS NSQIP database analysis by Vining *et al.* ^55^ found that the rate of clinically relevant (CR) POPF was higher in the OPD cohort compared to RPD, and that after propensity score matching, RPD was protective against CR-POPF. This has been confirmed in other studies, including a meta-analysis of four RCT and seventeen propensity score matched retrospective studies, which found that RPD had the highest probability of the best outcome for POPF grades B & C^48,53^. The same meta-analysis found that patient who underwent RPD had the highest probability of having the best outcome following PPH^48^. One study reports the rate of PPH in RPD at 4.8%, which is within range of most OPD series^56^. RPD has been associated with equivalent to improved rates of DGE when compared to OPD^46,48,57^.

### Laparoscopic approach versus robotic approach

While the literature shows improved LOS and LN harvest in RDP over LDP, few studies have directly compared RPD to LPD. Those that do find overall similar outcomes^38,48,58^. The most significant and important difference between RPD and LPD has been the rate of conversion. Across multiple studies, when compared with LPD, RPD is associated with a lower rate of conversion to open^38,59–61^. The RPD conversion rate ranges anywhere from as low as 4.1% in high volumes centres to 15–20% in medium to low volume centres^61,62^. Not only does conversion impact LOS and complication rate, but it has been shown that the need to convert to an open pancreatoduodenectomy increases the risk of patient morbidity and mortality^38^. More recent studies have aimed to dispel the concerns over conversion risk. A recent NCDB study showed that RPD resulted in a higher LN yield and shorter LOS even when converted to open, as compared to the open group. MIPD and MIPD cases that were converted to open were both significantly associated with increased likelihood of long-term survival when compared to OPD^63^.

### Learning curve for robotic pancreatoduodenectomy

Familiarity with RPD has been shown to improve outcomes^50,61,62^. Thus, as more centres implement RPD, it is essential that surgeons understand the learning curve associated with this procedure. Boone and colleagues reviewed 200 consecutive RPDs and identified several inflexion points that correspond to performance optimization at a high-volume RPD centre. After 20 RPDs, there was a significant improvement in estimated blood loss and conversion rate. After 40 cases the rate of POPF improved and more LNs were harvested. After 80 cases, operative time improved significantly. Quality analysis demonstrated that safety, efficiency and oncologic capability were optimized after the first 80 cases^64^. Studies have also shown that MIPD outcomes are not just dependent on individual surgeon operative volume but also hospital volume. Torphy *et al.* ^38^ demonstrated that patients who underwent surgery at a facility that performed >6 MIPD/year had decreased odds of 90-day mortality compared to those who had surgery at centres that performed fewer than 6 MIPD/year. International guidelines have recommend that these operations be performed at high-volume centres and future trials be conducted only with surgeons past their learning curve^8,9^. As the use of the robot becomes more widespread, these findings suggest that while many hospitals may be capable of offering RPD, this procedure should be limited to high-volume centres. It is important to note that OPD is the current standard of care and the predominate technique. Given the LEOPARD-2 trial and the decrease in LPD internationally, data from the three completed OPD versus RPD trials is eagerly anticipated. Most centres do not have the volume to consider opening MIPD programs. Very few training programs for MIPD exist and surgeons should not consider embarking on these procedures without adequate training^65,66^. In the US, review of the NCDB shows that most centres only perform one MIPD^37^. There is not causality in this data, but one assumption is it is abandoned after one case.

## What’s next

### Training

As the use of the robot becomes more common in pancreatic surgery it becomes essential to ensure surgeons are adequately trained in the technology. Research has shown that patients who have RDPs or RPDs performed by surgeons past their learning curve have better outcomes^32,33,50,64^. There is understandably concern over the risk to patients as surgeons work through their learning curves in robotic procedures^67^. However, robotic training curriculums provide a means to train surgeons in complex robotic techniques with minimal risk to patients. The Dutch Pancreatic Cancer Group developed a nationwide training program in MIDP and demonstrated decreased EBL, decreased conversion rate, and shorter LOS after completion of the curriculum^68^. Similar programs were subsequently created for LPD and RPD, and after completion of the training programs, surgeons met set benchmarks for low risk MIPD^69,70^. For RPD, an inflection point for operative time was found at 22 RPD procedures, which reflects a relatively short learning curve^70^. The Longitudinal Assessment and Realization of Minimally Invasive Pancreatic Surgery (LAELAPS) studies demonstrate that a multi-centre MIPS training program is a feasible way to train surgeons in these procedures with acceptable patient outcomes^68–70^. The LEARNBOT program aims to share these training curriculums with a broader European audience (http://e-mips.com/learnbot). Training in robotic surgery is equally essential at the resident and fellow level. Hogg *et al.* describes a mastery-based robotic simulation curriculum for surgical oncology fellows, who demonstrated dramatic improvement in their robotic skills^71^. Ongoing research has shown similar results at the resident level^72^.

### Ongoing trials

Any definitive conclusions regarding the benefits of robotic pancreatic surgery will be largely driven by randomized controlled trials. With the recent publication of the DIPLOMA trial, MIDP is now accepted as the preferred method to resect distal pancreatic malignancies by surgical oncologists^11^. However, the verdict is still out for MIPD, with ongoing randomized trials in this space. The international multi-centre “Minimally invasive versus open pancreatoduodenectomy for pancreatic and periampullary neoplasms” (DIPLOMA-2) trial just completed accrual and will compare overall complications and functional recovery to assess if MIPD (LPD or RPD) is superior to OPD for premalignant andmalignant pancreatic and periampullary disease (http://e-mips.com/diploma-2-trial)^73^. The “Robotic versus Open Pancreatoduodenectomy for Pancreatic and Periampullary Tumours” (PORTAL) trial from China is a multi-centre, phase III, non-inferiority trial where the primary outcome is time to functional recovery in RPD versus OPD^74^. These trials will be crucial in the wake of LEOPARD-2, and will help define the role of robotic pancreatoduodenectomy in the resection of pancreatic head and neck malignancies^42^.

### Artificial intelligence

Artificial intelligence (AI) is on the rise across medicine, with the goal of providing precise and individualized healthcare. One systematic review outlines the uses of AI in pancreas surgery, including in preoperative diagnosis of pancreatic masses from imaging studies, predicting patients at risk of intra-operative or postoperative complications, and identifying patients at increased risk of recurrence^75^. In robotic pancreas surgery, AI may be able to assist with surgical planning by identifying important structures and ideal dissection planes intraoperatively^76^. Although still in its early stages, the future of AI in pancreas surgery shows promise as a way to help surgeons provide the best care for their patients.

## Conclusion

This review serves to summarize the current literature regarding robotic pancreatic surgery for pancreatic cancer, with a focus on distal pancreatectomy and pancreatoduodenectomy. Since first performed in 2001, robotic pancreatic surgery has become increasingly popular. Still, the current research on this topic is largely limited to retrospective reviews, meta-analyses, and a small number of RCTs. The subset of research that focus on robotic pancreatic surgery for PDAC is smaller still.

Benefits seen with other types of robotic surgery, such as lower estimated blood loss and shorter LOS, are again seen in robotic pancreatic surgery. Some studies have started to associate RDP and RPD with equivalent oncologic outcomes as compared to open procedures, and even improved LN harvest. Furthermore, data suggest that the minimally invasive approach may improve the rate of certain complications, including POPF and DGE. However, one big question still remains: what is the impact of these robotic approaches on long-term oncologic outcomes and survival? Recently published RCTs have not been powered to draw definitive conclusions. As more surgeons are trained in minimally invasive pancreatic surgery and utilization of the robot in pancreatic surgery continues to increase, the answer will hopefully become clear.

## Ethical approval

Not applicable.

## Consent

Not applicable.

## Sources of funding

Not applicable.

## Author contribution

S.B.H.: conceptualization, writing—original draft, review and editing. A.E.R.: visualization—formation of figures, writing—review and editing. M.E.H.: conceptualization, resources, supervision, writing—review and editing.

## Conflicts of interest disclosure

Dr. Hogg has previously received funding for the institution from Intuitive^®^ for robotic training and education.

## Research registration unique identifying number (UIN)

Not applicable.

## Guarantor

Dr. Melissa E Hogg.

## Data statement

No datasets were generated or analyzed to write this review. Data sharing is not applicable to this article.

## Provenance and peer review

Invited.
